# Investigation of the impact of grain boundary hydrogen concentration on hydrogen embrittlement sensitivity of polycrystalline Fe: Molecular dynamics insights

**DOI:** 10.1002/smo2.70029

**Published:** 2025-12-17

**Authors:** Qiaoyun Tang, Wei Gao

**Affiliations:** ^1^ Department of Chemical Machinery and Safety Engineering State Key Laboratory of Fine Chemicals Dalian University of Technology Dalian China

**Keywords:** grain boundary, hydrogen atom concentration, hydrogen embrittlement sensitivity, molecular dynamics simulation

## Abstract

This study investigates the influence of hydrogen concentration at grain boundaries on the sensitivity of polycrystalline iron to hydrogen embrittlement using molecular dynamics simulations. These simulations reveal the diffusion behavior of hydrogen atoms at grain boundaries and their consequential impact on the hydrogen embrittlement sensitivity of iron alloys. The findings indicate that as the hydrogen concentration increases, both the yield strength and ultimate tensile strength of Fe‐H alloys exhibit a declining trend. Moreover, the capture of hydrogen atoms at the grain boundaries significantly influences the fracture toughness of the material and promotes the formation and propagation of cracks. This study provides a novel theoretical basis for understanding and predicting the hydrogen embrittlement behavior of iron‐based materials in hydrogen‐rich environments, offering valuable insights for the design and development of Fe alloys with enhanced resistance to hydrogen embrittlement.

## Graphical abstract

Molecular dynamics simulations were utilized to explore the influence of grain boundary hydrogen concentration on the hydrogen embrittlement sensitivity of polycrystalline iron. This graphical abstract visualizes the corresponding microstructural evolutions, with results demonstrating that elevated hydrogen concentration reduces the yield and ultimate tensile strengths of Fe‐H alloys, while grain boundary hydrogen capture facilitates crack formation and propagation—providing a theoretical basis for designing iron‐based materials with enhanced resistance to hydrogen embrittlement.

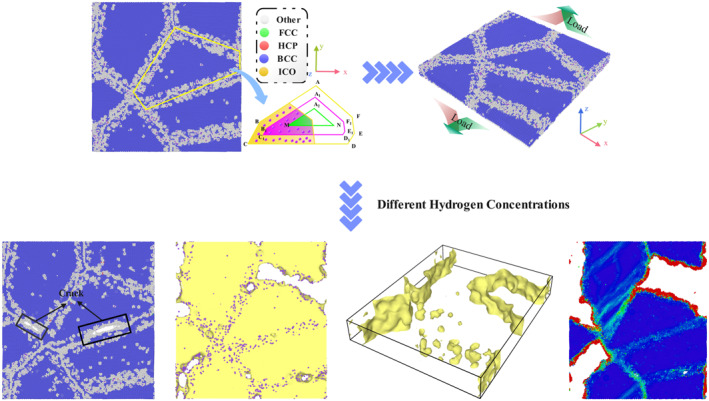

## 1 INTRODUCTION

During hydrogen storage and transportation, hydrogen atoms may infiltrate the pipeline material and accumulate at grain boundaries, leading to significant degradation of the material's mechanical properties and, consequently, triggering brittle fracture. The pipelines specifically designed for hydrogen transportation are crucial infrastructure for distributing hydrogen gas. Their safety and reliability are intrinsically linked to the effective utilization and consistent supply of hydrogen. Research indicates that hydrogen atoms can be generated through an adsorptive dissociation mechanism and subsequently penetrate steel pipelines, potentially inducing hydrogen embrittlement.^[1]^

Hydrogen embrittlement refers to the phenomenon where materials experience a significant reduction in toughness due to the permeation and accumulation of hydrogen atoms in a hydrogen‐rich environment, leading to brittle fracture at relatively low stresses. In hydrogen transportation pipelines, hydrogen atoms may penetrate the interior of the material through grain boundaries, lattice defects, and other pathways, accumulating at grain boundaries to form high‐concentration hydrogen traps, which significantly diminish the material's fracture toughness. In various environments, such as hydrocarbon compounds, aqueous solutions, and other corrosive chemical settings,[^2^, ^3^] the widespread presence of hydrogen atoms makes permeation a common issue. This phenomenon intensifies the vulnerability of metallic materials to hydrogen, especially in iron‐based alloys. The high diffusivity of active hydrogen makes these materials highly susceptible to brittle fracture under external stress, leading to a rapid deterioration in their mechanical properties.[^4^, ^5^, ^6^]

In general, the mechanical behavior of metals in hydrogen‐rich environments is primarily determined by metallurgical microstructures, such as the densities of reversible and irreversible hydrogen traps, inclusions, precipitates, and dislocations. These characteristics influence the diffusion and distribution of hydrogen atoms, thereby affecting the fatigue life of the pipelines. The interaction between hydrogen atoms and microstructural features further compromises the mechanical properties of metals, particularly affecting their fracture toughness and ductility. Several mainstream theories of the hydrogen embrittlement have been proposed based on the different physical processes and characteristics of metal‐hydrogen interactions.[^7^, ^8^, ^9^] In a study on metal fatigue crack propagation, Irwin et al.^[10]^ demonstrated that hydrogen‐rich environments accelerate fatigue crack propagation, prompting investigations into the accumulation of hydrogen within microcracks and voids in metallic materials. These findings revealed that hydrogen accumulated within these internal defects, creating high pressure that could drive crack propagation. Consequently, they proposed a hydrogen pressure theory. Katz et al.^[11]^ proposed the hydrogen‐enhanced decohesion embrittlement theory, based on the observation that hydrogen induces a transition from ductile to brittle fracture modes in certain metals. Beachem et al.^[12]^ studied the impact of hydrogen diffusion on dislocation motion in metals, proposing the hydrogen‐enhanced localized plasticity theory, which suggests that the accumulation of hydrogen atoms in metals can reduce the resistance to dislocation movement, thereby enhancing local plastic deformation and ultimately leading to crack formation and propagation. Furthermore, Sun et al.^[13]^ first identified the orientation dependence of this phenomenon. Lynch et al.^[14]^ proposed the hydrogen‐enhanced strain‐induced vacancy theory, which posits that the presence of hydrogen atoms can intensify the internal strain field within materials, inducing the formation of more vacancies and promoting the aggregation of hydrogen atoms at these sites, leading to the formation of microcracks and further causing brittle fracture. Wasim et al.^[15]^ studied the impact of hydrogen‐enhanced plasticity and decohesion mechanisms on the fracture resistance of steel, and proposed a model of their synergistic effects.

Early research primarily focused on comprehending the fundamental behavior of hydrogen within materials. Jothi et al.^[16]^ proposed a multi‐scale polycrystalline model to investigate the diffusion and trapping processes of hydrogen in polycrystalline materials, revealing the influence of microstructural characteristics on hydrogen embrittlement and providing theoretical support for understanding hydrogen embrittlement from a macroscopic perspective. Jung et al.^[17]^ conducted atomistic simulations to study the impact of hydrogen on the crack propagation behavior at grain boundaries in bcc iron, discovering that the tendency of hydrogen to segregate at grain boundaries is closely related to the grain boundary energy, offering direct evidence for understanding the micro‐mechanisms of hydrogen embrittlement. He et al.^[18]^ utilized first‐principles calculations to study the trapping and diffusion behavior of hydrogen in different grain boundary structures within γ‐iron, finding that hydrogen tends to segregate at certain grain boundaries, further refining the understanding of hydrogen behavior across various grain boundary types. The permeation behavior of hydrogen in 2205 duplex stainless steel was examined by Wu et al.^[19]^ from the perspective of practical use, revealing the important role that grain boundaries play in hydrogen transport. Ito et al.^[20]^ employed nano‐polycrystalline grain boundary models and molecular dynamics simulations to study the segregation behavior of hydrogen at grain boundaries in bcc iron polycrystals, noting that hydrogen tends to segregate at grain boundaries with segregation energies consistent with experimental data. Liu et al.^[21]^ expanded on this by using molecular dynamics simulations to investigate the segregation and fracture mechanisms of hydrogen at grain boundaries in magnesium crystals. They discovered that the critical energy release rate is influenced by the concentration of hydrogen and that the accumulation of hydrogen at grain boundaries considerably lowers the fracture toughness of the material. Furthermore, Xing et al.^[22]^ suggested that hydrogen accumulation at grain boundaries impedes both grain boundary and dislocation activity. Increased hydrogen concentrations may initiate microcracks at these interfaces. Additionally, the interaction between dislocations and grain boundaries increases local tensile stress, inducing intergranular fracture propagation.

Although existing research has advanced our understanding, significant limitations persist. Most studies have primarily examined specific materials or grain boundary types, lacking systematic comparisons of hydrogen behavior across different materials and grain boundary structures. Furthermore, limited understanding exists regarding specific hydrogen desorption sites at grain boundaries, hydrogen diffusion pathways, and their subsequent effects on macroscopic material properties. Consequently, this study investigates the influence of grain boundary hydrogen concentration on the hydrogen embrittlement susceptibility of the Fe atoms. This study clarifies the distribution, segregation, and diffusion behavior of hydrogen atoms at grain boundaries and how these behaviors impact the hydrogen embrittlement sensitivity of iron alloys using molecular dynamics simulations. It offers theoretical support and direction for the creation of Fe alloy materials with improved hydrogen embrittlement resistance.

## 2 MODELING AND SIMULATION DETAILS

**FIGURE 1:**
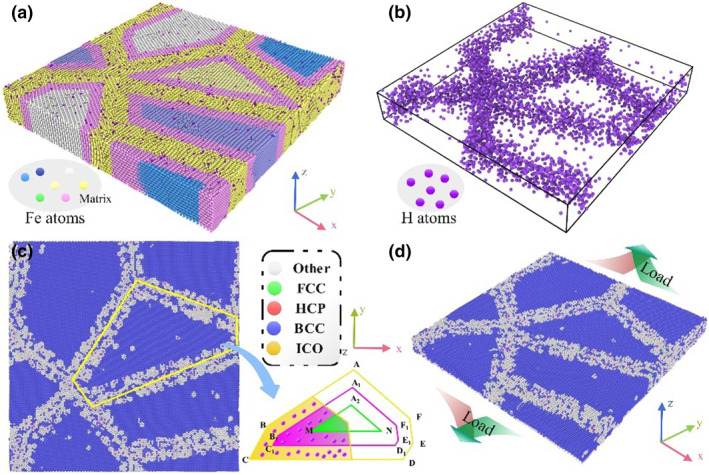
Fe‐H alloy model. (a) The distribution of hydrogen atoms within the polycrystalline Fe matrix; (b) the distribution of hydrogen atoms at grain boundaries after the removal of matrix atoms; (c) the microstructural distribution characterized by common neighbor analysis; (d) the direction of model loading.

To focus on the impact of hydrogen atoms on the mechanical behavior of materials, this study simplifies the hydrogen transportation pipeline material into a pure Fe alloy model. The LAMMPS is employed to theoretically investigate the micro‐deformation behavior of body‐centered cubic polycrystalline iron alloys at room temperature under varying hydrogen content captured at grain boundaries. Initially, a polycrystalline Fe matrix with four grains is created using Atomsk, with random rotation of grains to achieve orientational differences between grains. The model dimensions are set to 300 Å × 300 Å × 46 Å, encompassing approximately 340,000 atoms. The coordinates *x*, *y*, and *z* are aligned along the [100], [010], and [001] crystallographic directions, respectively. Overlapping atoms with distances less than 1.5 Å are removed from the model to ensure its initial stability. Iron atoms at grain boundaries (constructed based on the grain boundary influence zone concept proposed by Zhang^[23]^) are randomly replaced by hydrogen atoms at replacement rates of 1.6%, 7.1%, and 9.2%, as illustrated in Figure 1. By precisely controlling the number of hydrogen atoms substituted, this study simulates different hydrogen concentration conditions at grain boundaries.

**FIGURE 2:**
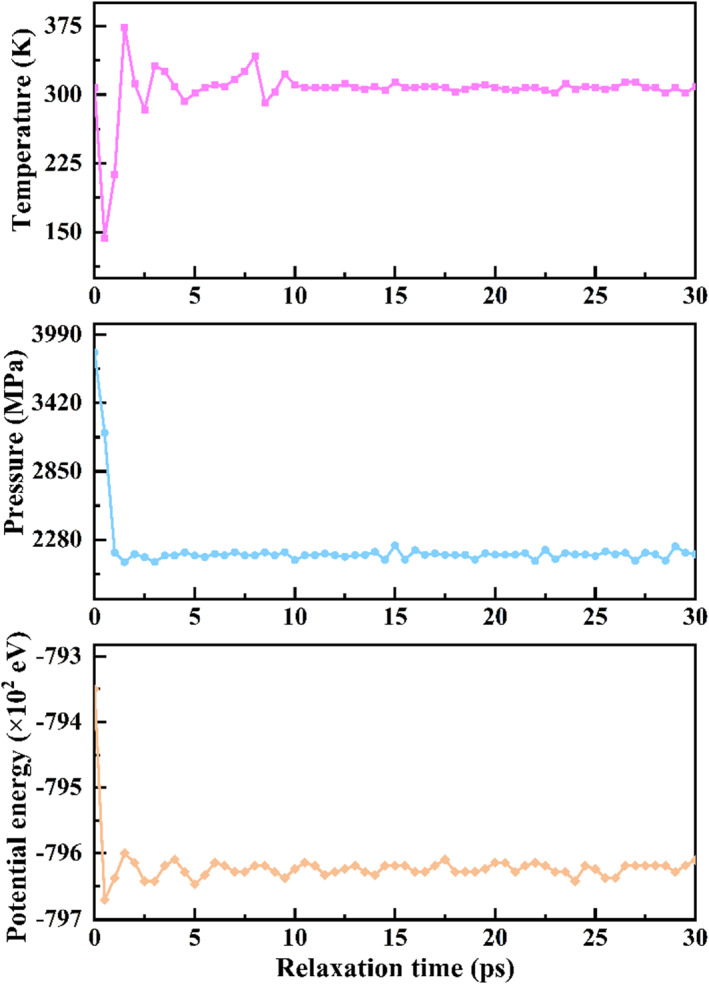
Curves of temperature, pressure, and potential energy with relaxation time.

Following the model construction, an energy minimization process was employed to optimize the atomic positions to ensure the initial stability of the system. The Fe‐Cr‐H embedded atom method potential, developed by Starikov,^[24]^ was utilized to describe the interatomic interactions within the Fe‐H system. Under an NVT ensemble at 300 K, the Nose‐Hoover thermostat was applied to equilibrate the polycrystalline Fe model for 300 ps. This step controlled the thermomechanical conditions of the sample and prevented the energy instability that might arise from temperature fluctuations. The conjugate gradient method was used to minimize the energy of the equilibrated model, with a timestep of 1 fs, to maintain the stability of the grain boundaries. Post‐equilibration, the changes in model temperature, pressure, and potential energy are shown in Figure 2, indicating that the model temperature and pressure reached the set values and the average potential energy of the atoms stabilized. Periodic boundary conditions were applied in all directions. The tensile process was accomplished by applying a strain rate of 0.001 Å/ps to a 1‐nm‐thick embedded layer along the *y*‐axis. During the loading period, the boundary conditions in the *x*‐direction of the system were extended, while those in the *y*‐direction were adjusted to an aperiodic setting. The OVITO was used to visualize and analyze the dynamic loading process of the model, graphically demonstrating the dimensional changes and internal structural evolution of the material during the tensile process. Common Neighbor Analysis (CNA)[^25^, ^26^] was employed to analyze the crystal structure, and the constructed surface mesh was used to observe the surface morphology and calculate the porosity rate. This approach not only enables a detailed analysis of the surface characteristics but also provides a quantitative assessment of the porosity under various conditions.

The tensile load was applied to the model sample along the *Y*‐axis direction through displacement loading. The procedure was detailed as follows: along the *Y*‐axis direction, four layers of atoms situated above and below the grain boundaries were designated as boundaries. Under loading, the lower four layers of atoms remain fixed, while the upper four layers of atoms undergo gradual displacement to induce stretching. The time step Δ*t* was set to 0.005 ps, the engineering strain rate of tensile loading was 1 × 10^5^ s^−1^, and the timestep in the calculation process was set to 1 fs. The strain produced in the system can be expressed as follows:

(1) $$\varepsilon =N\cdot \Delta t\cdot \frac{\varepsilon_{e}}{1\times 10^{12}}\times 100\%$$

wherein, *ε* represents the strain of the system; *N* denotes the number of timesteps for system simulation; Δ*t* signifies the timestep of system simulation; and *ε*_*e*_ represents the engineering strain rate. It can be calculated that the strain produced by the system is 20%, that is, the simulated system stops stretching when the strain is 20%.

## 3 SIMULATION RESULTS AND DISCUSSION

### 3.1 Effect of hydrogen atom concentration trapped on the grain boundary on the mechanical properties of polycrystalline Fe

**FIGURE 3:**
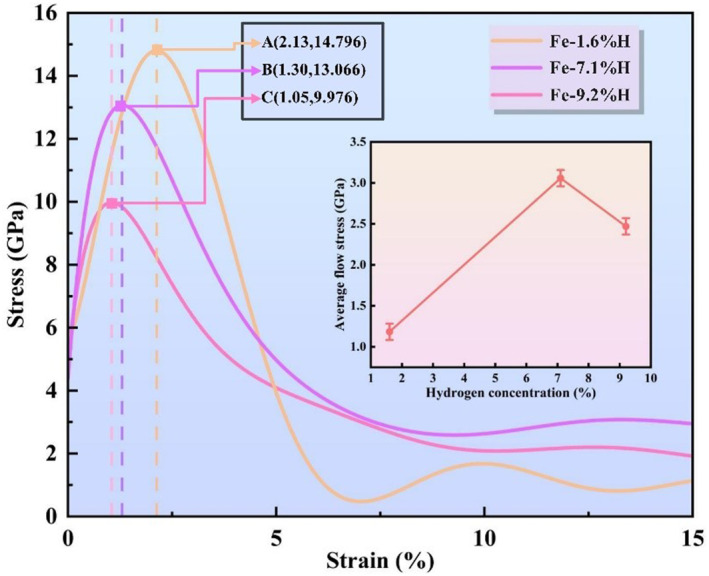
Stress‐strain curve of tensile deformation and average flow stress‐hydrogen concentration curve of nanocrystalline Fe alloys with different hydrogen atom contents.

To investigate the impact of varying hydrogen concentrations captured at grain boundaries on the mechanical properties of polycrystalline Fe, we analyze the stress‐strain curves of polycrystalline Fe obtained from uniaxial tension tests and the average flow stress within the strain range of 5%–15%, as depicted in Figure 3. For ease of identification and differentiation, the samples are designated as Fe‐1.6%H, Fe‐7.1%H, and Fe‐9.2%H, corresponding to the different hydrogen concentrations at the grain boundaries. As the hydrogen concentration increases from 1.6% to 9.2%, both the yield strength and ultimate tensile strength of Fe alloys exhibit a downward trend. This observation is consistent with hydrogen embrittlement theory, which suggests that the diffusion and aggregation of hydrogen atoms within the crystal lattice can impede dislocation motion, thereby reducing the material's ductility and leading to a decrease in strength. It is noteworthy that within a low strain range (*ε* ≤ 5%), the alloy with a hydrogen concentration of 1.6% demonstrates the highest stress level, indicating that within this strain range, the influence of lower hydrogen concentrations on strength is relatively minor. Further analysis reveals that as the hydrogen concentration increases, the ductility of the alloy decreases. This is evidenced by a reduction in the slope of the stress‐strain curve and a decrease in the area under the curve, signifying a reduction in the plastic deformation the material can sustain. In particular, the Fe‐9.2%H alloy exhibits a rapid stress drop after reaching its peak strength, indicating a lower ductility, which is consistent with the phenomenon of hydrogen atoms aggregating at grain boundaries, leading to grain boundary embrittlement.

Furthermore, the stress‐strain curves demonstrate that as the hydrogen concentration increases, the material's transition from ductile to brittle fracture is accelerated. This is consistent with hydrogen embrittlement theories, where the capture and aggregation of hydrogen atoms at grain boundaries promote transgranular or intergranular fracture modes. At hydrogen concentrations of 7.1% and 9.2%, the stress‐strain curves of the alloys exhibit lower ductility and higher brittleness, suggesting that higher hydrogen concentrations accelerate the brittle transition of the material. The trend of average flow stress with hydrogen concentration in Figure 3 further confirms the aforementioned analysis. As hydrogen concentration increases from 1.6% to 7.1%, the average flow stress shows an upward trend, peaking after which it begins to decline with further increase in hydrogen concentration. This indicates that there is an optimal value for hydrogen concentration on flow stress, beyond which hydrogen embrittlement effects become significant. Hydrogen concentration also affects strain hardening behavior. At low hydrogen concentrations, the alloy may exhibit certain strain hardening capabilities, whereas at high hydrogen concentrations, the strain hardening effect is diminished, and the fluctuation of the curve decreases. In summary, with the increase in hydrogen atom concentration captured at grain boundaries, the mechanical properties of Fe‐H alloys exhibit a trend of decreased strength, reduced ductility, and increased brittleness, aligning with predictions from hydrogen embrittlement theories.

As shown in Figure 3, the stress of the Fe‐H alloy initially shows a relatively higher stress level with increasing hydrogen concentration. However, when the strain exceeds 5%, the stress of the Fe‐1.6%H sample becomes significantly lower than that of the Fe‐7.1%H and Fe‐9.2%H samples. The abnormal stress variation phenomenon in Figure 3 can be attributed to the competitive effects of hydrogen segregation at grain boundaries, microdefect evolution, and typical hydrogen embrittlement mechanisms, which exhibit distinct characteristics at different hydrogen concentrations and strain stages: specifically, in the initial stage (*ε* ≤ 5%), hydrogen atoms in all samples preferentially segregate at grain boundaries due to its lower energy of grain boundaries—for Fe‐1.6%H sample, the limited hydrogen atoms strongly pin grain boundaries and inhibit dislocation emission and motion, resulting in a relatively higher initial stress; in contrast, for Fe‐7.1%H and Fe‐9.2%H samples, excessive hydrogen atoms not only segregate at grain boundaries but also occupy interstitial sites within grains, and the hydrogen‐enhanced local plasticity mechanism induced by intragranular hydrogen slightly promotes dislocation activity, leading to a slightly lower initial stress compared to the Fe‐1.6%H sample. When the strain exceeds 5%, the deformation mechanism shifts from dislocation motion to grain boundary sliding and crack initiation: for the Fe‐1.6%H sample, the low hydrogen content is insufficient to suppress crack propagation after grain boundary segregation occurs, and hydrogen‐induced grain boundary decohesion is triggered, causing rapid nucleation and growth of microcracks along grain boundaries and thus a sharp drop in stress; for the Fe‐7.1%H and Fe‐9.2%H samples, high hydrogen concentrations drive two competing effects—grain boundary segregation still induces decohesion, but intragranular hydrogen forms molecules or stabilizes vacancies, promoting dislocation multiplication and delaying crack coalescence. The latter effect dominates at high strains, allowing these samples to maintain higher stress before final fracture.

To determine Young's modulus for each model, the slope of each curve during the elastic stage was calculated. The peak stress (tensile strength) and the corresponding strain for each stress‐strain curve are also recorded, as shown in Table 1. These parameters serve to assess the strength and ductility of the polycrystalline Fe alloy, respectively, with higher values indicating better material properties. In the macroscopic experiments, the presence of numerous dislocations in the material typically results in lower stress levels compared to those of an ideal crystal. Consequently, variations in stress levels are primarily attributed to dislocations and slips. Therefore, the peak stress observed in this study is significantly higher than that observed in the macroscopic experiment.

**TABLE 1:** Essential parameters of mechanical properties for polycrystalline Fe models with different hydrogen atom concentrations at 300 K.

Hydrogen atom concentration (%)	Young's modulus (GPa)	Tensile strength (GPa)	Strain at maximum stress (%)
0	198.59	15.29	5.8
1.6	195.76	14.79	1.05
7.1	185.48	13.07	1.33
9.2	183.32	9.98	2.14

### 3.2 Effect of the concentration of hydrogen atoms trapped at grain boundaries on the evolution of micro‐defects in polycrystalline Fe

#### Hydrogen‐induced changes in crack propagation in polycrystalline Fe

**FIGURE 4:**
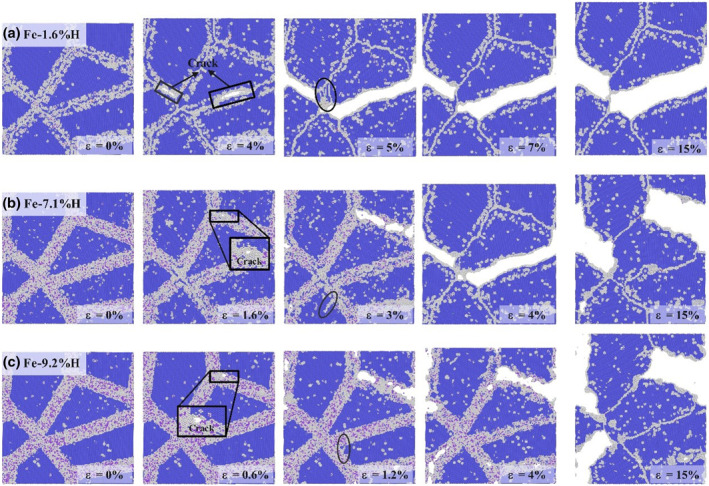
Under uniaxial tension, the atomic structure evolution diagram of Fe‐H alloy with different hydrogen atom content and external strain captured by grain boundaries is rendered by common neighbor analysis.

Figure 4 illustrates the evolution of polycrystalline Fe structures under uniaxial tension with different hydrogen concentrations at grain boundaries. These images reveal how hydrogen capture at grain boundaries affects material structural evolution and crack propagation behavior. Dislocation nucleation and crack initiation are key indicators for assessing material failure in tension tests. Observations show that in the Fe‐1.6%H, Fe‐7.1%H, and Fe‐9.2%H models, crack generation and propagation exhibit different sensitivities, with microcrack initiation strains of 4%, 1.6%, and 0.6%, respectively. Higher hydrogen concentrations lower the strain threshold for crack initiation, as cracks appear at lower applied strains in these models. In the Fe‐1.6%H model, cracks first appear at 4% strain, likely due to hydrogen aggregation at grain boundaries, which reduces boundary cohesive strength and promotes crack nucleation. At 5% strain, intense intergranular fracture suggests that hydrogen atoms further promote stress concentration at grain boundaries, accelerating crack propagation. As the strain increases to 15%, intergranular fracture worsens, ultimately causing complete fracture. This is attributed to continuous hydrogen aggregation at grain boundaries, reducing boundary cohesive strength, lowering fracture toughness, and making cracks more likely to propagate along grain boundaries. In the Fe‐7.1% H model, cracks appear at 1.6% strain, and cracks along grain boundaries expand and dislocations nucleate as the strain reaches 3%, with crack propagation further intensifying as strain increases. In the Fe‐9.2%H model, cracks emerge at 0.6% strain. As strain increases to 1.2%, new cracks form at multiple grain boundary locations, dislocations are observed within the grains (highlighted by elliptical frames), and cracks propagate along grain boundaries. When the strain increases to 15%, grain boundaries become finer and cracks continue to expand along grain boundaries, leading to separation between adjacent grains, though the model does not fracture completely. This indicates that at low hydrogen concentrations, hydrogen atoms may promote dislocation motion, enhance local ductility, cause stress concentration at grain boundaries, and trigger crack formation and propagation. Conversely, at higher hydrogen concentrations, the strain threshold for crack initiation decreases, and hydrogen aggregation at grain boundaries may impede dislocation motion, causing dislocation pile‐up at grain boundaries, increasing local stress concentration, and promoting crack growth along grain boundaries.

The aforementioned phenomena are consistent with the hydrogen‐enhanced decohesion embrittlement mechanism, where the diffusion and aggregation of hydrogen atoms within the crystal lattice can reduce the atomic bonding force at grain boundaries, thereby promoting decohesion embrittlement. Moreover, the aggregation of hydrogen atoms at grain boundaries may impede dislocation motion, leading to the accumulation of dislocations at grain boundaries and further increasing local stress concentration, which promotes crack formation. During the loading process, while the stress on the grains remains relatively constant, the stress concentration at grain boundaries increases, ultimately leading to fracture. The spring effect of grain boundaries allows them to absorb stress; hence, fine‐grained thermally affected zones with high grain boundary density exhibit greater buffering effects and are capable of absorbing greater strain energy.

#### Hydrogen‐induced changes in pore evolution in polycrystalline Fe

**FIGURE 5:**
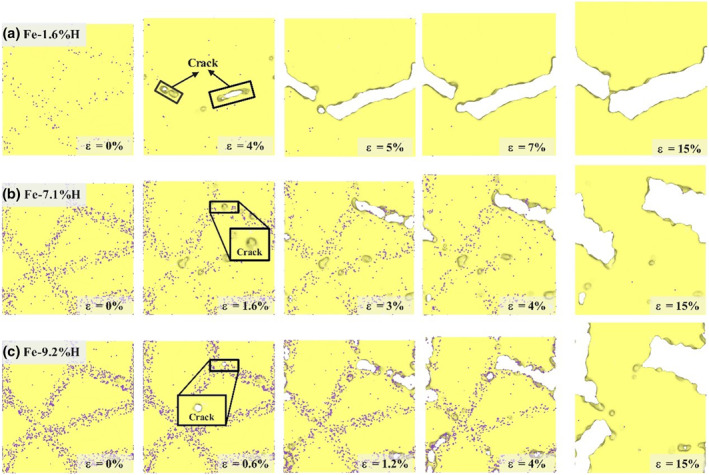
During uniaxial tension, H atom diffusion effect and surface morphology changes of Fe‐H alloy with different hydrogen atom content and external strain captured by grain boundaries.

To thoroughly observe the diffusion effect of hydrogen atoms captured at grain boundaries in polycrystalline Fe and its impact on surface morphology, we utilized the Construct Surface Mesh command in OVITO. This command identifies the atomic surface under the corresponding strain and intuitively displays the size and morphology of voids, as shown in Figure 5. The surface of polycrystalline Fe is rendered in yellow, with the boxed areas indicating voids and pores. The retention of hydrogen atoms at grain boundaries facilitates the observation of hydrogen atom capture at these boundaries. The capture and diffusion of hydrogen atoms at grain boundaries significantly affect the mechanical properties and surface morphology of the alloy. By using the Construct Surface Mesh command, it is evident that as the external strain increases, the diameter of voids along grain boundaries in each sample gradually increases and continuously expands along grain boundaries. Comparisons between Figure 5a–c reveal that the lower the hydrogen atom concentration at grain boundaries leads to more crack nucleation within the grains, making transgranular fracture more likely. In contrast, higher hydrogen concentrations at grain boundaries reduce the external strain needed for void nucleation. These observations indicate that the capture of hydrogen atoms at grain boundaries significantly affects the mechanical properties of polycrystalline Fe. It reduces fracture toughness and promotes crack formation and propagation. The presence of hydrogen atoms decreases the cohesive strength of grain boundaries, thereby promoting crack initiation. As the hydrogen concentration increases, the strain threshold for crack initiation decreases, making cracks more likely to grow along grain boundaries. Furthermore, the aggregation of hydrogen atoms at grain boundaries may hinder dislocation motion, leading to the accumulation of dislocations at grain boundaries, further increasing local stress concentration and promoting crack formation.

#### Hydrogen‐induced changes in porosity changes in polycrystalline Fe

**FIGURE 6:**
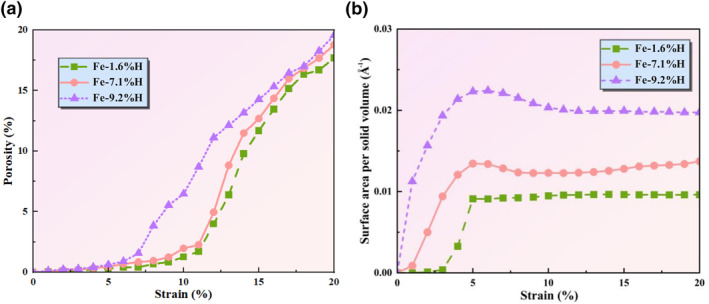
(a) The curves of porosity and (b) Surface area per solid volume of Fe‐H alloy under the influence of different hydrogen atom contents with external stress during uniaxial tension.

Porosity, as an indicator of the volume fraction of voids in a material relative to its total volume, is directly related to material density and significantly impacts mechanical properties. To visually assess the impact of hydrogen atom concentration captured at grain boundaries on the mechanical performance of Fe‐H alloys, we plotted the porosity curves as a function of strain, as shown in Figure 6a. The results show that porosity exhibits a clear upward trend with increasing strain, with varying growth rates under different hydrogen concentrations. Specifically, the alloy with a hydrogen concentration of 9.2% begins to exhibit significant porosity increase at a lower strain (approximately 5%), whereas the alloy with a lower hydrogen concentration (1.6%) requires a higher strain (approximately 10%) to achieve similar porosity growth. This phenomenon can be explained through the hydrogen embrittlement theory. Diffusion and aggregation of hydrogen atoms within the crystal lattice can reduce the atomic bonding force at grain boundaries, thereby promoting decohesion embrittlement. During the process of increasing strain, hydrogen atoms may aggregate at grain boundaries, increasing the brittleness of the boundaries and making the material more susceptible to crack propagation along grain boundaries, thus increasing porosity. Additionally, the presence of hydrogen atoms may impede dislocation motion, leading to the accumulation of dislocations at grain boundaries, further increasing local stress concentration and promoting crack formation and propagation. At low hydrogen concentrations, due to the smaller number of hydrogen atoms, their impact on material plastic deformation and crack propagation is relatively minor, thus requiring greater strain to induce significant porosity increase. In contrast, at high hydrogen concentrations, a larger number of hydrogen atoms are more likely to aggregate at grain boundaries, reducing the cohesive strength of the boundaries and causing the material to begin crack propagation at lower strains, leading to a rapid increase in porosity.

#### Hydrogen‐induced changes in surface area modifications in polycrystalline Fe

**FIGURE 7:**
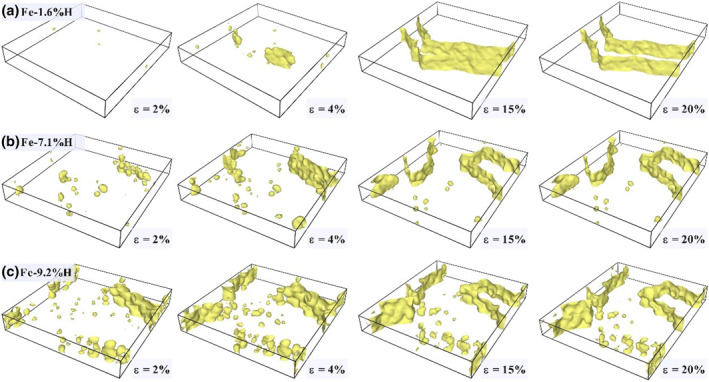
Variation trend of surface area of different hydrogen content models under uniaxial tension.

As shown in Figure 6a, the surface area to solid volume ratio of Fe‐1.6%H changes slightly with increasing strain, while that of Fe‐7.1%H and Fe‐9.2%H increases markedly. This indicates that higher hydrogen content leads to more significant surface area growth during tensile deformation, consistent with the observations in Figure 7. Under uniaxial tension, the surface area shows a gradual increasing trend with strain. Specifically, as strain increases from 2% to 20%, the surface area of pores and fracture surfaces in the models expands, with higher hydrogen content models exhibiting more significant growth. For instance, Fe‐1.6%H shows relatively small surface area changes, whereas Fe‐7.1%H and Fe‐9.2%H experience significant increases, especially at higher strains. The curve data in Figure 6b quantitatively support the phenomenon described in Figure 7, jointly revealing that the internal surface area of materials increases with higher hydrogen content and strain under uniaxial tension.

This phenomenon is mainly attributed to hydrogen embrittlement. Hydrogen atoms have high adsorption capacity at pores and fracture surfaces, reducing atomic cohesion and increasing material brittleness. As the strain increases, the defects and damage areas in the material expand, leading to an increase in the surface area of pores and fracture surfaces. Higher hydrogen content promotes more pronounced hydrogen diffusion and accumulation, accelerating the expansion of pores and fracture surfaces, and thus a more significant increase in surface area. Moreover, pores and fracture surfaces cause local stress concentration, which intensifies with increasing strain, further promoting their expansion. The presence of hydrogen exacerbates this stress concentration effect, making the material more susceptible to damage and fracture in these regions, thereby increasing the surface area.

### 3.3 Effect of hydrogen atom concentration trapped on grain boundary on local stress distribution of polycrystalline Fe

**FIGURE 8:**
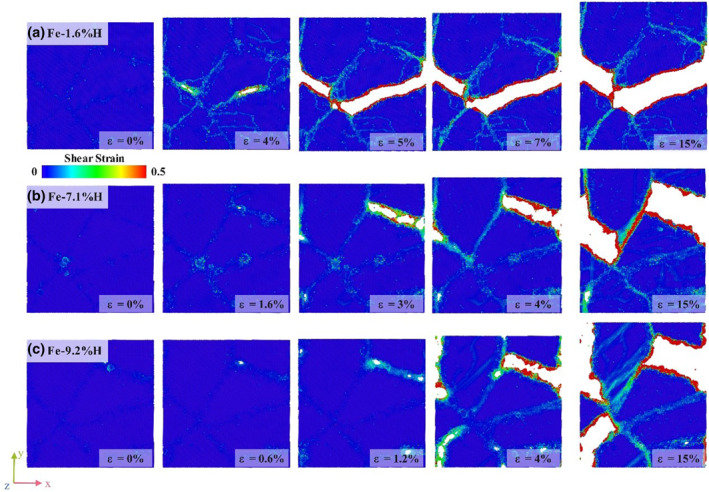
Distribution of atomic shear strain in Fe‐H alloy under the influence of different hydrogen atom content during uniaxial tension.

Figure 8 utilizes von Mises shear stress mapping to reveal the deformation characteristics of polycrystalline Fe at the atomic scale under varying hydrogen concentrations and applied strains, clearly depicting the local shear deformation in the phase transformation regions. Significant stress concentrations around voids can be observed, which are predominantly distributed in grain boundary areas with higher energy. Although the presence of a small amount of hydrogen atoms is insufficient to alter the body‐centered cubic structure as the primary plastic deformation mechanism, they do indeed facilitate the generation of dislocations during the initial stages of plastic deformation. The glide of these dislocations induces local plastic deformation around nanovoids and significantly alters the shape of the voids, increasing the likelihood of void growth and coalescence, ultimately leading to macroscopic hydrogen embrittlement.

Regarding mechanical response, the glide of dislocations necessitates higher shear stress. Consequently, polycrystalline Fe with higher hydrogen atom concentration exhibits lower critical stress values for triggering plastic deformation. This indicates that the presence of hydrogen atoms reduces the material's resistance to external loads, leading to plastic deformation at lower stress levels and accelerating the material's failure process. Furthermore, the distribution of shear stress under varying hydrogen concentrations in Figure 6 further confirms the embrittlement effect, where the increase in hydrogen concentration results in more stress‐concentrated regions within the material, which are more prone to dislocation activity and void formation, thereby triggering crack initiation and propagation at lower strain levels, consistent with the description in hydrogen embrittlement theories that hydrogen reduces the material's fracture toughness. Therefore, the capture of hydrogen atoms at grain boundaries not only affects the microstructure and mechanical behavior of polycrystalline Fe but also significantly increases the material's susceptibility to hydrogen embrittlement.

## 4 CONCLUSION

This study investigates the impact of grain ‐ boundary ‐ trapped hydrogen concentration on the hydrogen embrittlement sensitivity of polycrystalline Fe using molecular dynamics simulation. The results indicate that hydrogen atoms trapped at grain boundaries significantly affect the mechanical properties of polycrystalline Fe, reducing its fracture toughness and promoting crack formation and propagation. This research provides a theoretical basis for future studies on hydrogen behavior in different materials and its specific segregation sites and diffusion pathways at grain boundaries. The main conclusions are as follows:

(1) As hydrogen concentration increases, the yield strength and ultimate tensile strength of polycrystalline Fe decrease. This indicates that hydrogen diffusion and aggregation within the crystal lattice disrupt dislocation movement, thereby reducing the material's ability to undergo plastic deformation. The material also shows a stronger tendency to shift from ductile to brittle fracture, consistent with hydrogen embrittlement theory.
(2) Hydrogen capture and diffusion at grain boundaries significantly affect the alloy's surface morphology. With increasing strain, the pore diameter along grain boundaries grows and expands. Higher hydrogen concentrations lower the strain threshold for crack initiation.
(3) Hydrogen capture at grain boundaries significantly affects the porosity of polycrystalline Fe. Porosity increases with strain, but the rate differs with hydrogen concentration. Alloys with 9.2% hydrogen show a significant increase in porosity at around 5% strain, while those with 1.6% hydrogen require approximately 10% strain to achieve similar growth.
(4) The simulation results align with the hydrogen embrittlement theory. Hydrogen diffusion and aggregation in the lattice reduce the atomic bonding force at grain boundaries, promoting cleavage embrittlement. Hydrogen accumulation at grain boundaries may impede dislocation movement, causing dislocation pile‐up and increasing local stress concentration, thereby promoting crack formation.
(5) Hydrogen capture at grain boundaries affects the microstructure and mechanical behavior of polycrystalline Fe and increases its hydrogen embrittlement sensitivity. Hydrogen weakens grain boundary bonding and promotes crack initiation. With the increase in the concentration of hydrogen trapped at grain boundaries, cracks are more likely to propagate along grain boundaries.

## AUTHOR CONTRIBUTIONS

**Qiaoyun Tang:** Writing—original draft; methodology; investigation; formal analysis; data curation; conceptualization. **Wei Gao:** Writing—review and editing; supervision; resources; funding acquisition; data curation.

## CONFLICT OF INTEREST STATEMENT

The authors declare no conflicts interest.

## ETHICS STATEMENT

We promise that no animal or human experiments were involved in this study.

## Data Availability

No data were used in the research described in the article.
